# Cation‐Driven Valence Change Mechanism in 2D AgCrS_2_ for Ultralow‐Power and Reliable Memristors

**DOI:** 10.1002/advs.202521409

**Published:** 2026-02-04

**Authors:** Yueqi Su, Minghao Wang, Xiaolin Tai, Yuhua Liu, Yue Lin, Yuqiao Guo, Jing Peng, Yi Xie, Changzheng Wu

**Affiliations:** ^1^ Key Laboratory of Precision and Intelligent Chemistry CAS Center for Excellence in Nanoscience CAS Key Laboratory of Mechanical Behavior and Design of Materials Collaborative Innovation Center of Chemistry for Energy Materials (iChEM) and School of Chemistry and Materials Sciences University of Science and Technology of China Hefei China; ^2^ Institute of Energy Hefei Comprehensive National Science Center Hefei China

**Keywords:** 2D materials, cation‐driven valence change mechanism, planar memristors, reliable switching, ultralow power consumption

## Abstract

Memristive devices are promising building blocks for next‐generation memory and neuromorphic circuits in artificial intelligence. Among them, filamentary memristors offer great potential for high‐performance and densely integrated systems. However, achieving both low‐power operation and long‐term cycling stability remains a key challenge. Here, we present a 2D AgCrS_2_ volatile memristor that operates via a novel cation‐driven valence change mechanism (CVCM). Unlike traditional filament‐based conduction, this mechanism enables Ag^+^‐driven switching without metal filament growth. The threshold switching process is governed by the reversible intercalation of highly mobile Ag^+^ ions into tetrahedral vacancies between CrS_2_ layers, forming and rupturing the highly conductive Ag_2_CrS_2_ pathway and thus delivering an on/off ratio exceeding 10^5^ at 0.1 V. The AgCrS_2_ memristor enables a reduced threshold voltage of 0.2 V and an ultralow power consumption down to 200 pW when the compliance current is further reduced to the nA level. Additionally, the absence of elemental Ag metallization in the switching layer prevents structural degradation, enabling stable operation for over 3 × 10^5^ switching cycles. These findings establish CVCM as a promising way for developing energy‐efficient and reliable memristive technologies.

## Graphical abstract

A 2D AgCrS_2_ volatile memristor is shown to switch via a cation‐driven valence change mechanism, where Ag^+^ reversibly intercalates into tetrahedral vacancies between CrS_2_ layers to form a conductive Ag_2_CrS_2_ pathway without elemental Ag metallization. The device exhibits 0.2 V switching, nA‐compliance power down to 200 pW, and endurance beyond 3 × 10^5^ cycles, enabling energy‐efficient and stable neuronal elements.

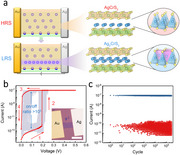

## 1 Introduction

Resistive switching devices, or memristors, have drawn intense interest as promising candidates for next‐generation memory and neuromorphic computing due to their nonlinear conduction characteristics and energy‐efficient processing capability [1, 2, 3]. Many mechanisms, such as Schottky barrier [4], space charge limited current [5] (SCLC) and filamentary conduction [6], have been proposed to explain the resistive switching effect in various memristive systems. Among them, filamentary conduction has been extensively investigated owing to its excellent performance and superior integration potential [6, 7, 8, 9]. Specifically, filamentary conduction involves the formation and rupture of conductive filaments within switching materials through field‐induced ionic migration [10]. This process is analogous to the synaptic Ca^2+^ dynamics in biological synapses [11, 12], offering fast switching between the high‐resistance state (HRS) and the low‐resistance state (LRS) with a large on/off ratio [12, 13]. While filamentary memristors exhibit these compelling features, further efforts are still required to meet the demands for practical applications, especially in minimizing power consumption and enhancing operational stability [8, 14].

According to the chemical composition of filaments, filamentary conduction can be classified into two categories: electrochemical metallization (ECM) and valence change mechanism (VCM) [15, 16, 17]. In ECM‐based memristors, resistive switching is realized by the growth and fracture of conductive metallized filaments between electrodes [18, 19]. By judiciously selecting highly mobile electrochemically active cations (e.g., Ag^+^ or Cu^+^), memristors based on ECM typically exhibit ultralow SET voltages (V_SET_, the voltage of abrupt switching from HRS to LRS) [20, 21, 22], making them highly attractive for energy‐efficient memory and computing applications. However, the uncontrolled formation of metallic filaments often induces irreversible structural damage to the switching layer, which significantly degrades the endurance and stability of the devices [23, 24, 25]. Although recent volatile ECM‐type devices (diffusive memristors) based on thin HfO_2_ films have demonstrated improved cycling endurance [13, 17], the stochastic metallization/demetallization process still raises concerns about long‐term structural robustness and device‐to‐device variability, especially for 2D planar systems [26]. Alternatively, VCM typically employs migration of oxygen or sulfur anions, creating conductive filaments through local changes in the stoichiometry of the switching material [27, 28]. Although VCM circumvents the issue of structural damage caused by metallization [29], the inherently high migration barriers of anions result in elevated V_SET_ and power consumption, which severely limit their practical applications [30, 31, 32]. Additional efforts have been made to address these limitations, for example, by introducing functional layers to regulate the cation transport [33], or by building a 3D vertical structure to achieve simultaneous control over both cations and anions [34]. Nonetheless, such strategies typically introduce additional complexity into device fabrication processes, and the ambiguous ionic migrations also raise reliability concerns. Therefore, a fundamental solution that integrates the advantages of ECM and VCM while overcoming their intrinsic limitations in device reliability and power consumption is highly desired for memristors based on filamentary conduction.

Recently, memristors based on layered 2D materials have been found to effectively regulate ionic migration through interlayer confinement [35, 36]. Here, we demonstrated a novel cation‐driven valence change mechanism (CVCM) in 2D AgCrS_2_ volatile memristors. Through reversible migration of external Ag^+^ into intrinsic tetrahedral sites between the CrS_2_ layers, the growth and rupture of conductive channels are achieved due to local variations in compositional stoichiometry rather than the formation of elemental Ag filaments, leading to threshold resistive switching with a high switching ratio exceeding 10^5^ at a read voltage of 0.1 V. Upon lowering the applied voltage below the HOLD voltage (V_HOLD_, the voltage below which the device relaxes back to HRS), the non‐equilibrium Ag^+^‐rich configuration relaxes and drives Ag^+^ back‐diffusion toward the Ag reservoir, rupturing the conductive channel and restoring the HRS. Unlike conventional VCM devices, where the anion diffusion typically requires a relatively large activation energy [32] (>1 eV), highly mobile Ag^+^ cations give rise to a significantly reduced V_SET_ of 0.2 V and thus an ultralow power consumption of 200 pW with a compliance current of 1 nA. Additionally, in contrast to ECM‐based systems, the inserted Ag^+^ cations remain confined within the vacant tetrahedral sites without forming metallized filaments, thereby effectively avoiding irreversible structural degradation and enhancing device endurance (> 3 × 10^5^ cycles). This CVCM paradigm successfully combines the low‐voltage and energy‐efficient characteristics of ECM with the structural robustness and high reliability of VCM, providing valuable insights for developing filamentary memristors with superior performance, excellent power efficiency, and good device stability for next‐generation memory and neuronal elements applications.

## 2 Results and Discussion

**FIGURE 1:**
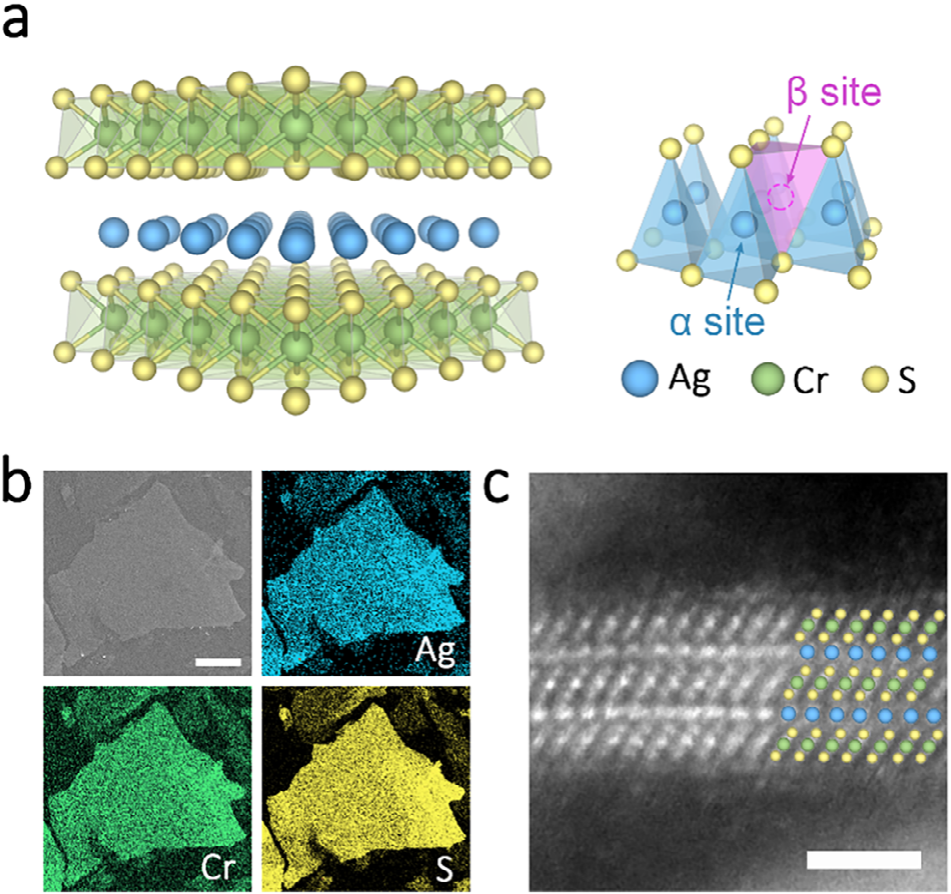
Characterizations of AgCrS_2_ nanosheets. a) Crystal structure of the AgCrS_2_ monolayer. The Ag^+^ ions occupy half of the equivalent tetrahedral sites (α and β sites) between two CrS_2_ octahedral slabs. b) The SEM image and the Ag, Cr and S mapping of AgCrS_2_ nanosheets. Scale bar, 30 µm. c) Cross‐section HAADF‐STEM image of a AgCrS_2_ bilayer sample. Scale bar, 1 nm. Atom color code in (a) and (c): Ag, blue; Cr, green; S, yellow.

AgCrS_2_ crystallizes in a hexagonal structure with Ag^+^ ions occupying half of the equivalent tetrahedral sites (α and β sites) between CrS_2_ octahedral layers [37] as illustrated in Figure 1a. By using a redox‐controlled strategy [38], AgCrS_2_ crystal can be exfoliated down to a monolayer with a thickness of 1.2 nm (Figure S1). The 2D AgCrS_2_ nanosheets exhibit high quality and a similar lattice structure to that of the bulk material, which was confirmed by Raman spectroscopy (Figure S2) and high‐resolution transmission electron microscopy (HRTEM) images (Figure S3). A spatially homogeneous elemental distribution across the flake was validated by electron probe microanalysis (EPMA) mapping (Figure 1b), and further quantitative analysis (Figure S4) indicated a stoichiometric formula of Ag_n_Cr_n+1_S_2(n+1)_ (n = 1, 2, 3…, where n is the number of Ag layers) for the nanosheets of different thicknesses. Cross‐section high‐angle annular dark‐field scanning transmission electron microscopy (HAADF‐STEM) in Figure 1c reveals the atomic structure of a typical bilayer AgCrS_2_ nanosheet, in which two Ag layers are alternately inserted between three CrS_2_ slabs.

**FIGURE 2:**
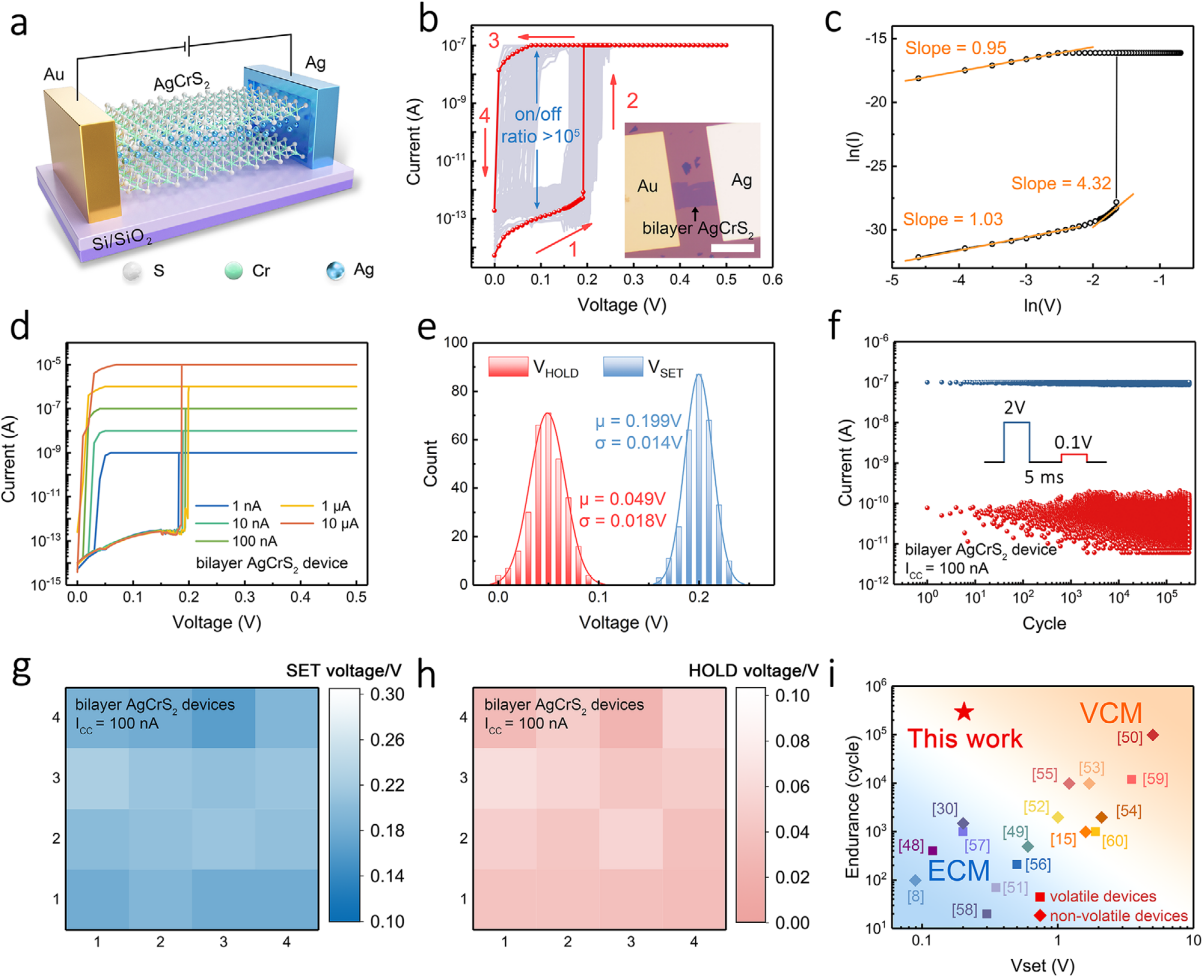
Electrical behaviors of planar Au/AgCrS_2_/Ag devices. a) Schematic of a planar Au/AgCrS_2_/Ag device. b) I‐V switching curves of a bilayer AgCrS_2_ device for 300 consecutive cycles measured with I_CC_ = 100 nA. The red arrows indicate the sweep directions. The inset shows the optical image of the device. Scale bar, 10 µm. c) ln(I)‐ln(V) plots with linear fittings, indicating trap‐mediated SCLC behavior in the HRS and Ohmic conduction in the LRS. d) Switching behaviors of the bilayer AgCrS_2_ device measured under different I_CC_ values from 10 µA to 1nA. e) Distributions of V_SET_ and V_HOLD_ extracted from (b) with Gaussian fits, where µ is the mean value and σ is the standard deviation. f) Pulse endurance test for the bilayer AgCrS_2_ device measured with I_CC_ = 100 nA, showing stable switching up to 3 × 10^5^ cycles. As shown in the inset, the LRS and HRS of the device were read at 2 and 0.1 V, respectively. g,h) Device‐to‐device variability of V_SET_ (g) and V_HOLD_ (h) for 16 individually fabricated bilayer AgCrS_2_ devices. i) Comparison of SET voltages and endurance among representative filamentary memristors.

Considering that the inherent tetrahedral vacancies between CrS_2_ layers can serve as convenient channels for the anisotropic transport of mobile active metal cations [39, 40], AgCrS_2_ emerges as a promising solid electrolyte material for memristors with high performance. To evaluate its resistive switching behavior, Au/AgCrS_2_/Ag devices were fabricated with a planar architecture. Figure 2a displays the structure schematic of a standard device, in which the active Ag metal electrode serves as the source of Ag^+^ cations while the inert Au electrode is grounded. Figure 2b presents the representative current‐voltage (*I–V*) characteristics of a bilayer AgCrS_2_ device measured for 300 consecutive cycles with a compliance current (I_CC_) of 100 nA, revealing clear threshold switching behavior. Specifically, at a positive voltage around 0.2 V, the device undergoes an abrupt transition from HRS to LRS, and spontaneously relaxes back to HRS as the voltage returns to zero. At a read voltage of 0.1 V, the device exhibits a switching ratio exceeding 10^5^, which is remarkably high for planar memristors [41]. The I‐V curves were further plotted in a double logarithmic scale. As can be seen in Figure 2c, the current follows a linear I∝V relationship at low bias and a power‐law relationship with I∝V^4.32^ at a higher voltage in the HRS, consistent with the typical trap‐mediated SCLC model [42, 43]. In the LRS, the experimental curve follows the Ohmic behavior, which is similar to the transport features of the reported Ag‐based filamentary memristors [44, 45].

To examine the operating conditions relevant to low‐power switching, we further measured the same bilayer device under different current compliances from 10 µA down to 1 nA (Figure 2d). Remarkably, even at a low I_CC_ of 1 nA, the device still exhibits clear and stable threshold resistive switching behavior with an extremely low SET voltage of 0.2 V, which leads to an estimated power consumption as low as 200 pW by using the formula of P = I_CC_×V_SET_. In addition, thickness‐dependent measurements (Figure S5) show that the V_SET_ decreases systematically with reducing AgCrS_2_ thickness and reaches 0.15 V in the monolayer limit with I_CC_ = 10 µA. This trend is consistent with previous reports that the activation energy for Ag^+^ migration decreases as the layer number of AgCrS_2_ nanosheets is reduced [38]. Looking forward, scalable CVD growth [46, 47] and transfer of ultrathin AgCrS_2_ could enable more uniform large‐area integration while further lowering the switching power under appropriately limited compliance.

The switching stability and uniformity of the AgCrS_2_ devices were also evaluated. Cycle‐to‐cycle statistical analysis extracted from the 300 *I–V* cycles in Figure 2b shows narrow distributions of V_SET_ and V_HOLD_ (Figure 2e), indicating highly reproducible and well‐controlled threshold switching behavior within the same device. Remarkably, under pulsed operation with I_CC_ = 100 nA, the bilayer device exhibits stable switching up to 3 × 10^5^ cycles (Figure 2f; Figure S6), demonstrating excellent endurance as an Ag‐based memristor. In addition, we also assessed device‐to‐device variability by characterizing 16 individually fabricated devices under identical measurement conditions. As summarized in Figure 2g,h, both V_SET_ and V_HOLD_ exhibit relatively narrow spreads across the device population, supporting good uniformity at the device level. Finally, we benchmarked the SET voltage and endurance of the AgCrS_2_ devices against representative filamentary memristors (Figure 2i). Overall, the combination of low‐voltage operation and high cycling stability of the AgCrS_2_ memristor compares favorably with conventional VCM‐ and ECM‐based filamentary devices [8, 15, 30, 48, 49, 50, 51, 52, 53, 54, 55, 56, 57, 58, 59, 60], rendering it highly promising for neuronal elements in neuromorphic circuits where both energy efficiency and switching reliability are critical.

**FIGURE 3:**
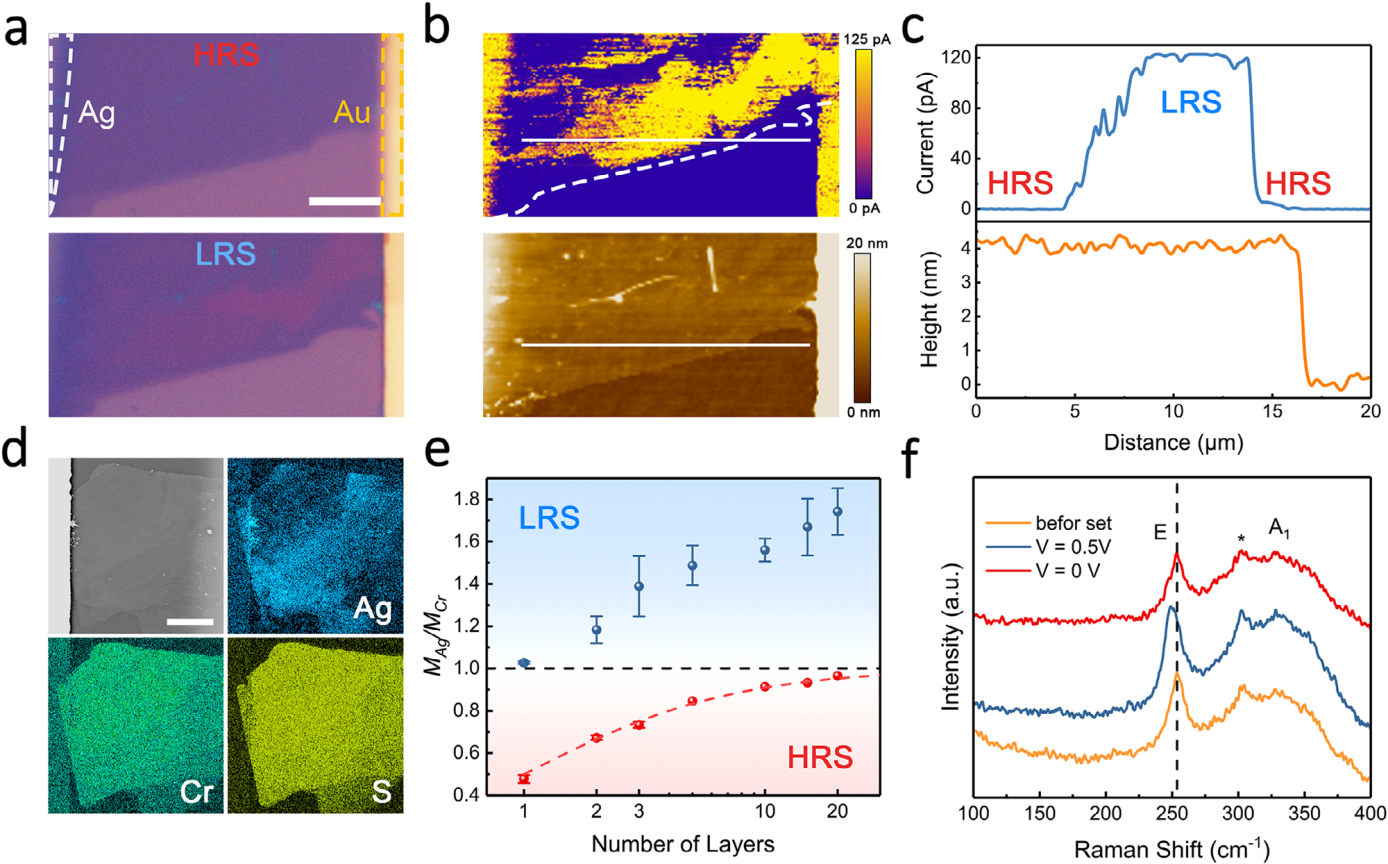
Characterizations of the planar Au/AgCrS_2_/Ag device during switching. a) Optical images of the same device in the HRS (top) and LRS (bottom). The dashed outlines indicate the boundary of Ag and Au electrodes. Scale bar, 5 µm. b) Current map (top) and topographic image (bottom) of the device in the LRS from (a) by CAFM characterizations. The dashed outline indicates the boundary of the AgCrS_2_ nanosheet. c) Current and height profiles along the white lines in (b). d) The SEM image and the Ag, Cr and S mapping of the device in the LRS. Scale bar, 10 µm. e) Molar ratio of Ag/Cr for the devices in the HRS (red) and LRS (blue) based on AgCrS_2_ nanosheets with varying thicknesses. The red dashed line indicates the theoretical ratio for original AgCrS_2_ nanosheets. f) In situ Raman spectra for the device during switching. The asterisk‐marked peak is assigned to the silicon substrate.

To investigate the underlying threshold switching mechanism, in situ optical characterizations were performed on planar AgCrS_2_ devices. Distinct from traditional ECM‐based memristors, which necessarily involve the formation and rupture of metal filaments [6, 13], there is no evidence of metallized Ag filaments on the AgCrS_2_ nanosheets during the switching process, according to the optical microscopy (OM) and scanning electron microscopy (SEM) analysis in Figure 3a,d. Instead, a significant change in color from purple to red was observed in specific regions of the AgCrS_2_ nanosheet when the device was switched to LRS, as can be seen in Figure 3a. In detail, as the bias reached V_SET_, the color‐switched zone spread swiftly from the Au side toward the Ag electrode (Video S1), which is very similar to the growth pattern of conductive Ag filaments [61, 62], implying a common origin arising from the migration of Ag^+^ cations. As the optical properties of 2D materials are highly related to their band structures, changes in the color of nanosheets can reflect variations in their electrical properties [63]. Indeed, once the colorized zone bridges both electrodes, the device is switched from HRS to LRS, suggesting that this region may serve as a conductive channel across the insulating AgCrS_2_ sheet.

To further confirm the filamentary switching in AgCrS_2_ memristors, conductive atomic force microscopy (CAFM) was employed to provide a detailed local electronic characterization. Different from vertical devices, which require precise etching to remove the top electrode [64], the planar AgCrS_2_ memristor with an inherently exposed resistive switching layer facilitates the CAFM characterization, as shown in Figure S7. The device was switched into LRS with a high voltage of 2 V to ensure it remained in the LRS long enough, and then a reading bias of 0.1 V between the Ag electrode and the Pt/Ir probe was applied during the CAFM test. According to the current map shown in Figure 3b, we noticed a clear conducting path when the device was set to LRS, confirming the formation of a localized conductive pathway during the resistive switching in AgCrS_2_. Remarkably, there exists a precise spatial congruence between the conductive region and the colorized zone observed in Figure 3a, which substantiates the conducting nature of the color‐switched zone. This correspondence enables macroscopic visualization of the growth behavior of conductive pathways using conventional OM, thereby providing a straightforward, non‐invasive method for real‐time monitoring of the switching process. The CAFM analysis also confirmed that the surface of the AgCrS_2_ nanosheet remains smooth and uniform with no sign of metallized Ag filaments even after the device being switched to LRS. Profiles comparing the height and current in Figure 3c validated that the conductivity enhancement in the nanosheet does not accompany any topographical expansion or contraction. These findings suggest that the resistive switching is unlikely to originate from ECM, but may instead arise from a redistribution of ions within the lattice framework, as in VCM.

EPMA was utilized to study the elemental distribution and quantitatively analyze the composition of AgCrS_2_ nanosheets in different resistance states. According to the elemental mapping in Figure 3d, Cr and S were uniformly distributed across the sample when the device was switched into LRS. However, the distribution of Ag was highly inhomogeneous with significantly higher concentrations localized in the regions of enhanced local conductivity (Figure S8). Further quantitative analysis reveals that the molar ratio of Ag/Cr in these regions exceeded the theoretical value, reaching up to twice the expected value (Figure 3e). Given that AgCrS_2_ contains two sets of tetrahedral interstices between CrS_2_ layers and the initial Ag^+^ ions occupied only half of them, it is proposed that additional Ag^+^ ions are intercalated into the vacant tetrahedral sites. This intercalation induces a structural transition from CrS_2_−Ag−CrS_2_ to CrS_2_−Ag_2_−CrS_2_, corresponding to a doubling of Ag^+^ layers between CrS_2_ slabs. For multilayer samples, the molar ratio of Ag/Cr in locally conducting regions was found to range approximately from 1 to 2n/(n+1) (n = 1, 2, 3…, where n is the number of initial Ag layers), indicating that full occupation of at least one additional Ag layer within the interstitial sites is necessary to achieve the LRS. Such stoichiometric changes in the composition of nanosheets imply an underlying CVCM in AgCrS_2_ memristors. Notably, the Au/AgCrS_2_/Au device with both inert electrodes shows no evidence for resistive switching (Figure S9), which confirms that the observed switching is not driven by the migration of oxygen or sulfur anions as in conventional VCM‐based memristors, but instead originates from a cation‐driven process involving Ag^+^ intercalation.

In addition, both CAFM and EPMA maps show that the conducting region expands more broadly on the Au electrode side than the Ag electrode side, indicating a higher local Ag^+^ concentration near the Au side. Once the applied voltage falls below the hold voltage, the external electric field is no longer sufficient to sustain such a non‐equilibrium Ag^+^‐rich configuration. The relaxation of the local Ag^+^ electrochemical potential, together with the associated internal electromotive field arising from the Ag^+^ concentration gradient, drives partial back‐diffusion of Ag^+^ ions toward the Ag reservoir [65]. As a result, the Ag_2_CrS_2_ pathway is fragmented into discontinuous conducting regions (Figure S10), and the device returns to the macroscopic HRS while exhibiting a slightly elevated HRS current compared to the initial cycle (Figure 2b). Also, the intercalation and extraction of Ag^+^ cations during the RS process do not induce any detectable structural degradation, which was confirmed by in situ Raman spectroscopy measurements shown in Figure 3f and Figure S11. The two identified Raman peaks at 250 cm^−1^ and 325 cm^−1^ correspond to the in‐plane (E) and out‐of‐plane (A_1_) vibrational modes, respectively [37, 38]. The negligible change in the peak profiles suggests that the lattice structure of the nanosheets remains intact throughout the switching process. Notably, there is a reversible redshift in the E peak as the device is switched into LRS, which is likely due to enhanced electron‐phonon scattering and is often observed in 2D materials intercalated by molecules or ions that lead to electron doping [66, 67, 68].

**FIGURE 4:**
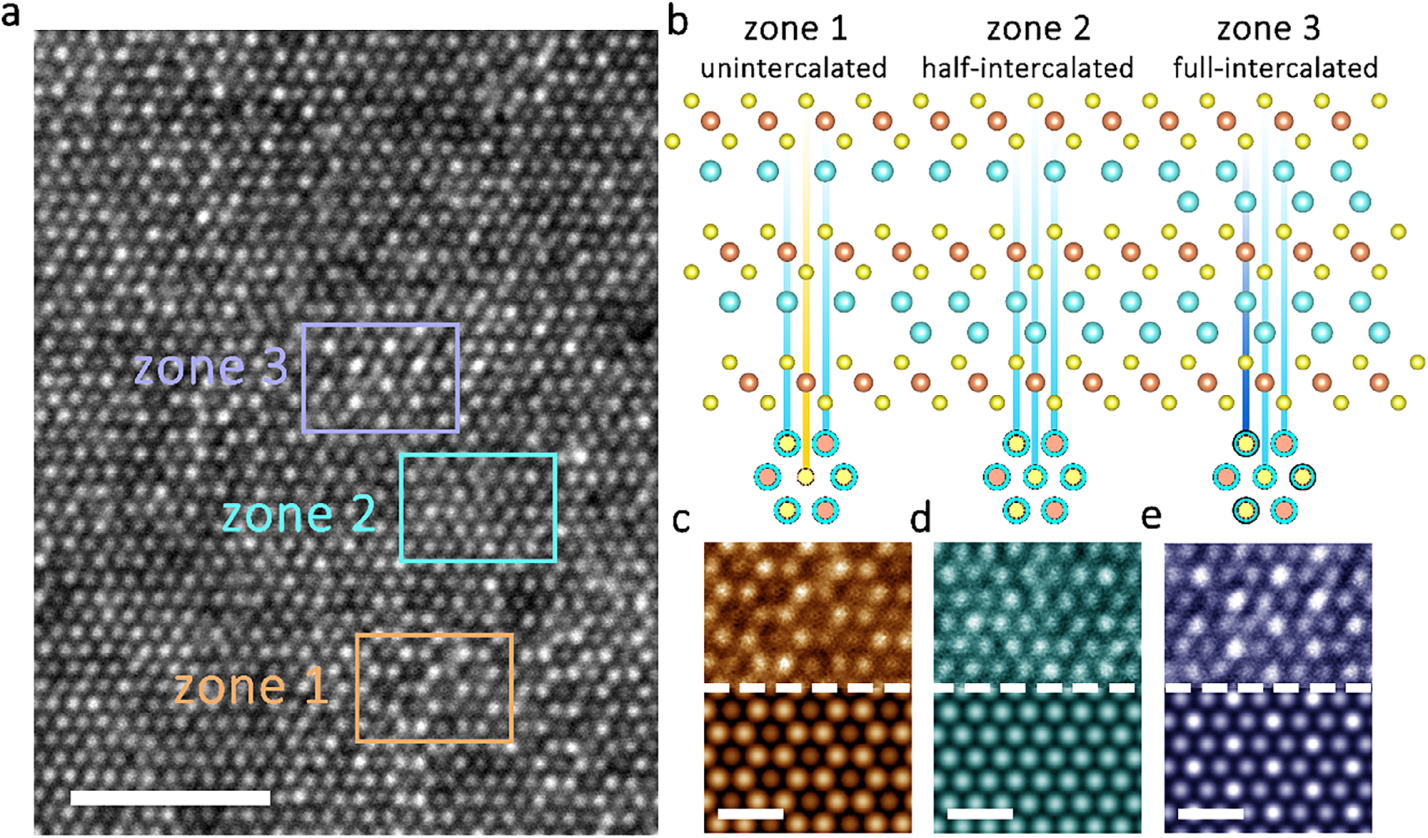
Silver ion distribution in the conductive Ag_2_CrS_2_. a) HAADF‐STEM image of a bilayer sample along the [001] direction from a device switched to the LRS. Scale bar, 2 nm. b) Cross‐view of the AgCrS_2_ in different stages of intercalation by external Ag^+^ ions and the corresponding top‐view. c–e) Top: Magnified images of the zones 1, 2, and 3 in (a), respectively. Bottom: Simulated HAADF‐STEM top‐view images of AgCrS_2_ bilayer in the unintercalated (c), half‐intercalated (d), and full‐intercalated (e) states, respectively. Scale bar, 0.5 nm. Atom color code in (b): Ag, cyan; Cr, orange; S, yellow.

To further explore the distribution of Ag^+^ cations within nanosheets, HAADF‐STEM was employed to characterize the locally conducting regions of a bilayer AgCrS_2_ nanosheet sample. As shown in Figure 4a, the HAADF‐STEM top‐view images unveiled a hexagonal grid pattern along the [001] direction with good crystallinity, demonstrating that the intercalation of Ag^+^ cations does not disrupt the lattice integrity nor induce elemental Ag metallization, maintaining the structural stability of the nanosheets. Interestingly, three distinct zones with varying patterns were observed in the top‐view images. As can be seen in Figure 4c–e, Zone 1 displayed a honeycomb pattern, where each dark site was surrounded by six bright sites, consistent with the theoretical simulations of the pristine bilayer AgCrS_2_ nanosheets with no intercalation of external Ag^+^ cations. In contrast, most of the sample resembled Zone 2, where the brightness of each site is uniform. Theoretical simulations indicate that this pattern corresponds to half‐intercalated bilayer AgCrS_2_, where a single layer of vacant tetrahedral sites is occupied by external Ag^+^ cations, as illustrated in Figure 4b. As for Zone 3, it exhibited significantly brighter sites, suggesting an increase in the local concentration of Ag^+^ ions, which is consistent with a pattern of full‐intercalated AgCrS_2_. The HAADF‐STEM observations correlate well with the EPMA quantitative analysis, confirming that the intercalated Ag^+^ cations are confined within the non‐van der Waals gaps of the 2D AgCrS_2_, occupying the vacant tetrahedral sites and forming a new structure of Ag_2_CrS_2_ without generating elemental Ag filaments. This structural transition not only preserves the crystalline integrity but also enhances the electrical conductivity, corroborating the formation of a highly conductive pathway within the nanosheet.

**FIGURE 5:**
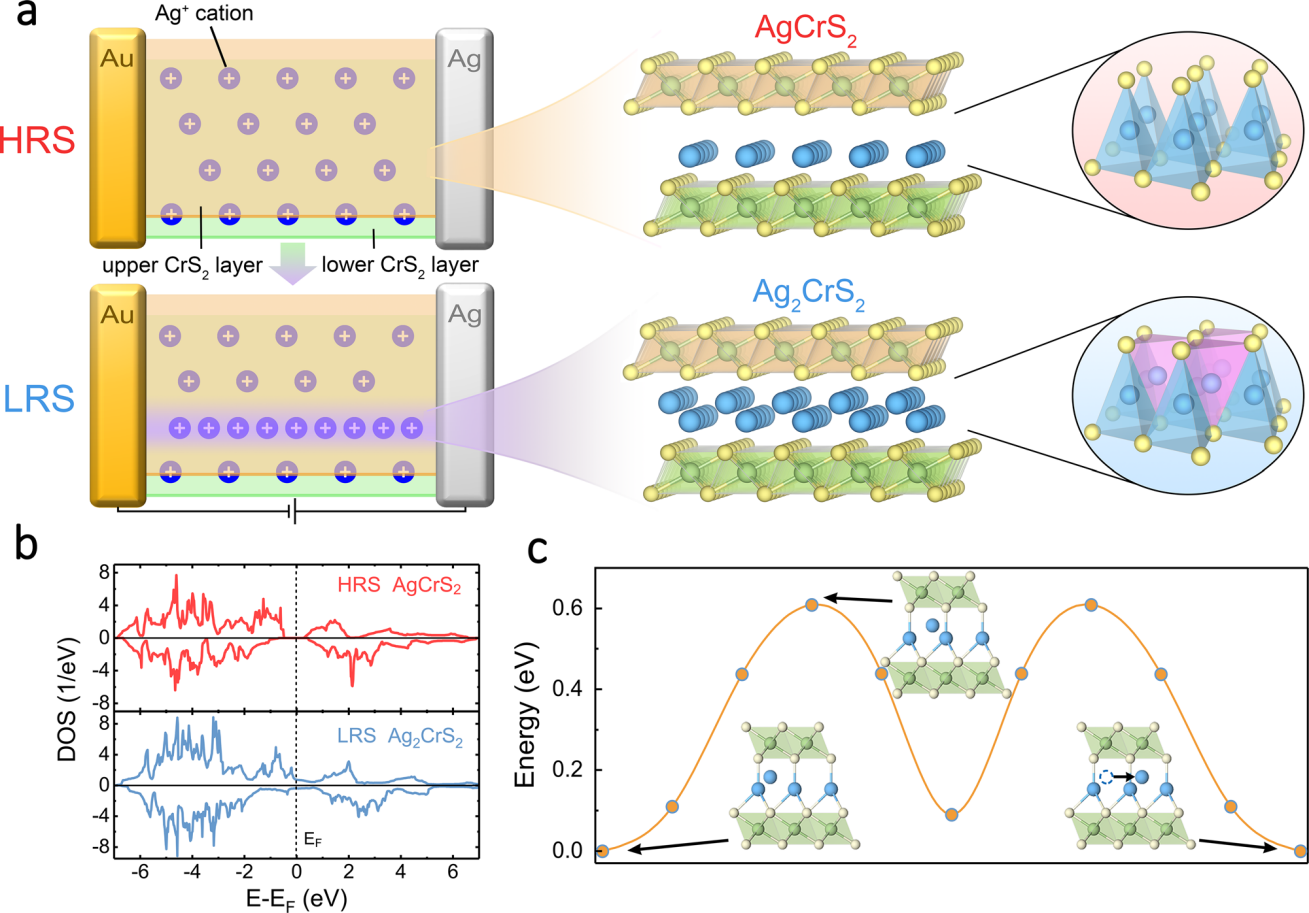
Theoretical calculations. a) Schematic illustration of the CVCM. The left panel shows the migration of external Ag^+^ ions from the Ag electrode toward the Au electrode, while the right panel illustrates the structural transition from AgCrS_2_ to Ag_2_CrS_2_. b) Calculated DOS of AgCrS_2_ (top) and Ag_2_CrS_2_ (bottom). c) The ionic migration landscape for monolayer Ag_1.25_CrS_2_. The insets show the position of the external Ag^+^ ion along the migration path. Atom color code in (a) and (c): Ag, blue; Cr, green; S, yellow.

To analyze the CVCM in AgCrS_2_, we calculated the density of states (DOS) of AgCrS_2_ and Ag_2_CrS_2_ with different compositional stoichiometries (Figure 5a). As illustrated in Figure 5b, pristine AgCrS_2_ is a semiconductor with a bandgap of 0.8 eV. Upon full intercalation of Ag^+^ ions into the empty tetrahedral sites, the system undergoes a semiconductor‐to‐metal transition, resulting in a gapless metallic DOS for Ag_2_CrS_2_. This electronic transition is consistent with the experimentally observed conductivity enhancement during the switching process. To further understand the switching dynamics, we computed the migration barrier of Ag^+^ ions in a partially intercalated monolayer Ag_1.25_CrS_2_. The ionic migration path connects two adjacent tetrahedral β sites (Figure 5c), which happens in the transition between HRS and LRS. The calculated energy barrier is 0.61 eV, which is remarkably low compared to migration barriers of oxygen or sulfur anions in typical memristors based on VCM [32] and explains the observed low SET voltage in the device. The structural stability of monolayer Ag_2_CrS_2_ was verified via ab initio molecular dynamics (AIMD) simulations as shown in Figure S12, demonstrating its robustness under room‐temperature conditions. These results demonstrate the CVCM as an underlying origin of the observed resistive switching behavior. Driven by an external bias, the intercalated Ag^+^ ions undergo low‐barrier migration and reversible intercalation into the vacant tetrahedral sites between CrS_2_ layers instead of forming elemental Ag filaments, leading to a stoichiometric transition from semiconducting AgCrS_2_ to conductive Ag_2_CrS_2_ and thereby enabling switching. When the applied voltage drops below the HOLD voltage, the absence of a sustaining electric field allows the local Ag^+^ electrochemical potential to relax and drives back‐diffusion of Ag^+^ ions toward the Ag reservoir, which breaks the continuity of the Ag_2_CrS_2_ pathway and restores a high‐resistance configuration.

## 3 Conclusion

We have demonstrated a novel cation‐driven valence change mechanism (CVCM) in the filamentary resistive switching of AgCrS_2_ memristors. Through electrochemical intercalation of Ag^+^ cations into intrinsic tetrahedral vacancies between the CrS_2_ layers, highly conductive Ag_2_CrS_2_ pathways are formed within the semiconducting AgCrS_2_ nanosheets, giving rise to threshold switching with a high on/off ratio exceeding 10^5^. Benefiting from highly mobile Ag^+^ ions, the cation‐driven resistive switching achieves a reduced SET voltage of 0.2 V and an ultralow power consumption of 200 pW with I_CC_ = 1 nA, significantly outperforming memristors based on anion‐driven VCM. Meanwhile, different from the ECM process, the inserted Ag^+^ cations remain confined within the tetrahedral vacancies without elemental Ag metallization, thereby maintaining structural integrity and enabling excellent endurance exceeding 3 × 10^5^ cycles. These results highlight the CVCM as a new compelling paradigm for the rational design of next‐generation memristive systems that simultaneously exhibit excellent performance, ultralow power consumption, and high operational reliability.

## Author Contributions

This project was supervised and directed by J.P., C.W. and Y.X. J.P. and Y.S. conceived this work. Y.S. designed the experiments. Y.S., M.W. and Y. Liu conducted the device fabrication and electrical measurements. Y.S. and M.W. performed the material characterizations. X.T. and Y. Lin carried out the STEM experiments. M.W. conducted the density functional theory calculation. Y.S., M.W., X.T. and Y.G. conducted the data analyses. Y.S. and J.P. co‐wrote the manuscript. All authors contributed to the discussion and analysis of the results.

## Conflicts of Interest

The authors declare no conflicts of interest.

## Supporting information




**Supporting File 1**: advs73952‐sup‐0001‐SuppMat.docx.

**Supporting File 2**: advs73952‐sup‐0002‐VideoS1.mp4.

## Data Availability

The data that support the findings of this study are available from the corresponding author upon reasonable request.

## References

[1] M. A. Zidan , J. P. Strachan , and W. D. Lu , “The Future of Electronics Based on Memristive Systems,” Nature Electronics 1 (2018): 22–29.

[2] A. Sebastian , M. L. Gallo , R. Khaddam‐Aljameh , and E. Eleftheriou , “Memory Devices and Applications for In‐Memory Computing,” Nature Nanotechnology 15 (2020): 529–544. 10.1038/s41565-020-0655-z 32231270

[3] K. X. Sun , J. S. Chen , and X. B. Yan , “The Future of Memristors: Materials Engineering and Neural Networks,” Advanced Functional Materials 31 (2021): 2006773.

[4] V. K. Sangwan , H. S. Lee , H. Bergeron , et al., “Multi‐Terminal Memtransistors From Polycrystalline Monolayer Molybdenum Disulfide,” Nature 554 (2018): 500–504. 29469093 10.1038/nature25747

[5] A. A. Gismatulin , G. N. Kamaev , V. N. Kruchinin , V. A. Gritsenko , O. M. Orlov , and A. Chin , “Charge Transport Mechanism in the Forming‐Free Memristor Based on Silicon Nitride,” Scientific Reports 11 (2021): 2417. 33510310 10.1038/s41598-021-82159-7 PMC7843651

[6] A. A. Bessonov , M. N. Kirikova , D. I. Petukhov , M. Allen , T. Ryhanen , and M. J. A. Bailey , “Layered Memristive and Memcapacitive Switches for Printable Electronics,” Nature Materials 14 (2015): 199–204. 25384168 10.1038/nmat4135

[7] M. Lanza , H. S. P. Wong , E. Pop , et al., “Recommended Methods to Study Resistive Switching Devices,” Advanced Electronic Materials 5 (2019): 1800143.

[8] Y. Liu , X. F. Zhou , H. Yan , et al., “Highly Reliable Textile‐Type Memristor by Designing Aligned Nanochannels,” Advanced Materials 35 (2023): 2301321. 10.1002/adma.202301321 37154271

[9] C. Yoon , G. Oh , S. Kim , et al., “Implementation of Threshold‐ and Memory‐Switching Memristors Based on Electrochemical Metallization in an Identical Ferroelectric Electrolyte,” NPG Asia Materials 15 (2023): 33.

[10] Q. Liu , J. Sun , H. B. Lv , et al., “Real‐Time Observation on Dynamic Growth/Dissolution of Conductive Filaments in Oxide‐Electrolyte‐Based ReRAM,” Advanced Materials 24 (2012): 1844–1849. 22407902 10.1002/adma.201104104

[11] H. B. Zhou , S. F. Li , K. W. Ang , and Y. W. Zhang , “Recent Advances in In‐Memory Computing: Exploring Memristor and Memtransistor Arrays With 2D Materials,” Nano‐Micro Letters 16 (2024): 121. 38372805 10.1007/s40820-024-01335-2 PMC10876512

[12] Z. R. Wang , S. Joshi , S. E. Savel'ev , et al., “Memristors With Diffusive Dynamics as Synaptic Emulators for Neuromorphic Computing,” Nature Materials 16 (2017): 101–108. 27669052 10.1038/nmat4756

[13] R. Midya , Z. R. Wang , J. M. Zhang , et al., “Anatomy of Ag/Hafnia‐Based Selectors With 10 10 Nonlinearity,” Advanced Materials 29 (2017): 1604457. 10.1002/adma.201604457 28134458

[14] T. Ahmed , S. Kuriakose , S. A. Tawfik , et al., “Mixed Ionic‐Electronic Charge Transport in Layered Black‐Phosphorus for Low‐Power Memory,” Advanced Functional Materials 32 (2022): 2107068.

[15] J. Y. Chen , C. W. Huang , C. H. Chiu , Y. T. Huang , and W. W. Wu , “Switching Kinetic of VCM‐Based Memristor: Evolution and Positioning of Nanofilament,” Advanced Materials 27 (2015): 5028–5033. 26193454 10.1002/adma.201502758

[16] D. S. Jeong and C. S. Hwang , “Nonvolatile Memory Materials for Neuromorphic Intelligent Machines,” Advanced Materials 30 (2018): 1704729. 10.1002/adma.201704729 29667255

[17] Z. R. Wang , H. Q. Wu , G. W. Burr , et al., “Resistive Switching Materials for Information Processing,” Nature Reviews Materials 5 (2020): 173–195.

[18] Y. C. Yang , F. Pan , Q. Liu , M. Liu , and F. Zeng , “Fully Room‐Temperature‐Fabricated Nonvolatile Resistive Memory for Ultrafast and High‐Density Memory Application,” Nano Letters 9 (2009): 1636–1643. 19271714 10.1021/nl900006g

[19] Y. C. Yang , P. Gao , S. Gaba , T. Chang , X. Q. Pan , and W. Lu , “Observation of Conducting Filament Growth in Nanoscale Resistive Memories,” Nature Communications 3 (2012): 732. 10.1038/ncomms1737 22415823

[20] P. X. Lei , H. Duan , L. Qin , et al., “High‐Performance Memristor Based on 2D Layered BiOI Nanosheet for Low‐Power Artificial Optoelectronic Synapses,” Advanced Functional Materials 32 (2022): 2201276.

[21] X. Y. Guo , Q. Wang , X. W. Lv , et al., “SiO_2_ /Ta_2_O_5_ heterojunction ECM Memristors: Physical Nature of their Low Voltage Operation With high stability And Uniformity,” Nanoscale 12 (2020): 4320–4327. 32043511 10.1039/c9nr09845c

[22] M. Jang , H. C. Song , H. J. Kim , J. H. Yoon , and K. M. Kim , “Multielement Filament Memristor Enabling Multifunctional Neuromorphic Device,” Advanced Functional Materials 35 (2025): 2423273.

[23] H. B. Lv , X. X. Xu , H. T. Liu , et al., “Evolution of Conductive Filament and its Impact on Reliability Issues in Oxide‐Electrolyte Based Resistive Random Access Memory,” Scientific Reports 5 (2015): 7764. 25586207 10.1038/srep07764 PMC4293596

[24] S. K. Acharya , J. Jo , N. V. Raveendra , et al., “Brownmillerite Thin Films as Fast Ion Conductors for Ultimate‐Performance Resistance Switching Memory,” Nanoscale 9 (2017): 10502–10510. 28708191 10.1039/c7nr04011c

[25] J. H. Yoon , J. M. Zhang , P. Lin , et al., “A Low‐Current and Analog Memristor With Ru as Mobile Species,” Advanced Materials 32 (2020): 1904599. 10.1002/adma.201904599 31984587

[26] G. L. Ding , R. S. Chen , P. Xie , et al., “Filament Engineering of Two‐Dimensional h ‐BN for a Self‐Power Mechano‐Nociceptor System,” Small 18 (2022): 2200185. 10.1002/smll.202200185 35218611

[27] S. Munjal and N. Khare , “Valence Change Bipolar Resistive Switching Accompanied With Magnetization Switching in CoFe_2_O_4_ Thin Film,” Scientific Reports 7 (2017): 12427. 28963521 10.1038/s41598-017-12579-x PMC5622061

[28] M. Wang , S. H. Cai , C. Pan , et al., “Robust Memristors Based on Layered Two‐Dimensional Materials,” Nature Electronics 1 (2018): 130–136.

[29] J. J. Yang , M. X. Zhang , J. P. Strachan , et al., “High Switching Endurance in TaOx Memristive Devices,” Applied Physics Letters 97 (2010): 232102.

[30] Y. S. Li , Y. Xiong , X. L. Zhang , et al., “Memristors With Analogue Switching and High On/Off Ratios Using a Van Der Waals Metallic Cathode,” Nature Electronics 8 (2025): 36–45.

[31] B. S. Tang , H. Veluri , Y. D. Li , et al., “Wafer‐Scale Solution‐Processed 2D Material Analog Resistive Memory Array for Memory‐Based Computing,” Nature Communications 13 (2022): 3037. 10.1038/s41467-022-30519-w PMC9160094 35650181

[32] R. Nakamura , T. Toda , S. Tsukui , et al., “Diffusion of oxygen in amorphous Al_2_O_3_, Ta_2_O_5_, and Nb_2_O_5_ ,” Journal of Applied Physics 116 (2014): 033504.

[33] X. F. Wang , H. Tian , H. M. Zhao , et al., “Interface Engineering With MoS_2_ –Pd Nanoparticles Hybrid Structure for a Low Voltage Resistive Switching Memory,” Small 14 (2018): 1702525. 10.1002/smll.201702525 29205799

[34] M. C. Wu , Y. H. Ting , J. Y. Chen , and W. W. Wu , “Low Power Consumption Nanofilamentary ECM and VCM Cells in a Single Sidewall of High‐Density VRRAM Arrays,” Advanced Science 6 (2019): 1902363. 31890465 10.1002/advs.201902363 PMC6918122

[35] H. Y. Qin , Z. J. Wang , Q. R. Li , et al., “Interstitial Ag+ Engineering Enables Superior Resistive Switching in Quasi‐2D Halide Perovskites,” Nanomaterials 15 (2025): 1267. 40863847 10.3390/nano15161267 PMC12389324

[36] B. Zhao , X. Zhao , X. C. Xun , et al., “Ion Intercalation‐Mediated MoS2 Conductance Switching for Highly Energy‐Efficient Memristor Synapse,” Advanced Electronic Materials 11 (2025): 2400633.

[37] P. Bruesch , T. Hibma , and W. Buhrer , “Dynamics of Ions of the Two‐Dimensional Superionic Conductor AgCrS_2_ ,” Physical Review B 27 (1983): 5052–5061.

[38] J. Peng , Y. H. Liu , H. F. Lv , et al., “Stoichiometric Two‐Dimensional Non‐Van Der Waals AgCrS_2_ With Superionic Behaviour at Room Temperature,” Nature Chemistry 13 (2021): 1235–1240. 10.1038/s41557-021-00800-4 34663918

[39] J. Peng , Y. H. Liu , Y. Pan , et al., “Fast Lithium Ion Conductivity in Layered (Li–Ag)CrS_2_ ,” Journal of the American Chemical Society 142 (2020): 18645–18651. 32902961 10.1021/jacs.0c08448

[40] G. R. Akmanova , N. N. Bikkulova , and A. D. Davletshina , “Two‐dimensional Superionic Conductors CuCrS_2_ and AgCrS_2_ and Their Alloys,” Russian Journal of Electrochemistry 49 (2013): 827–830.

[41] Z. P. Xia , X. Sun , Z. L. Wang , J. L. Meng , B. Y. Jin , and T. Y. Wang , “Low‐Power Memristor for Neuromorphic Computing: From Materials to Applications,” Nano‐Micro Letters 17 (2025): 217. 40227506 10.1007/s40820-025-01705-4 PMC11996751

[42] Z. Y. Lv , Y. Wang , Z. H. Chen , et al., “Phototunable Biomemory Based on Light‐Mediated Charge Trap,” Advanced Science 5 (2018): 1800714. 30250806 10.1002/advs.201800714 PMC6145401

[43] V. A. Voronkovskii , V. S. Allev , A. K. Gerasimova , and D. R. Islamov , “Conduction Mechanisms of TaN/HfO x /Ni Memristors,” Materials Research Express 6 (2019): 076411.

[44] X. B. Yan , J. H. Zhao , S. Liu , et al., “Memristor With Ag‐Cluster‐Doped TiO_2_ Films as Artificial Synapse for Neuroinspired Computing,” Advanced Functional Materials 28 (2018): 1705320.

[45] K. Y. Wang , L. T. Li , R. J. Zhao , et al., “A Pure 2H‐MoS_2_ Nanosheet‐Based Memristor With Low Power Consumption and Linear Multilevel Storage for Artificial Synapse Emulator,” Advanced Electronic Materials 6 (2020): 1901342.

[46] X. Xu , Y. X. Chen , P. B. Liu , et al., “General Synthesis Of Ionic‐Electronic Coupled Two‐Dimensional Materials,” Nature Communications 15 (2024): 4368. 10.1038/s41467-024-48690-7 PMC11111738 38778090

[47] J. B. Xing , Y. Tang , J. X. Li , et al., “Intrinsic Out‐Of‐Plane and In‐Plane Ferroelectricity in 2D AgCrS2 with High Curie Temperature,” Advanced Materials 36 (2024): 2407655. 10.1002/adma.202407655 39104282

[48] Q. Y. Li , Q. Y. Tao , Y. Chen , et al., “Low Voltage and Robust InSe Memristor Using Van Der Waals Electrodes Integration,” International Journal of Extreme Manufacturing 3 (2021): 045103.

[49] Y. Y. Shi , X. H. Liang , B. Yuan , et al., “Electronic Synapses Made of Layered Two‐Dimensional Materials,” Nature Electronics 1 (2018): 458–465.

[50] A. Prakash , S. Maikap , H. C. Chiu , T. C. Tien , and C. S. Lai , “Enhanced Resistive Switching Memory Characteristics and Mechanism Using a Ti Nanolayer at the W/Tao X Interface,” Nanoscale Research Letters 9 (2014): 125. 24636463 10.1186/1556-276X-9-125 PMC3995362

[51] M. C. Sahu , A. K. Jena , S. K. Mallik , et al., “Reconfigurable Low‐Power TiO_2_ Memristor for Integration of Artificial Synapse and Nociceptor,” ACS Applied Materials & Interfaces 15 (2023): 25713–25725. 37199948 10.1021/acsami.3c02727

[52] F. M. Simanjuntak , T. Ohno , S. Chandrasekaran , T. Y. Tseng , and S. Samukawa , “Neutral Oxygen Irradiation Enhanced Forming‐Less Zno‐Based Transparent Analog Memristor Devices For Neuromorphic Computing Applications,” Nanotechnology 31 (2020): 26LT01. 10.1088/1361-6528/ab7fcf 32168495

[53] D. L. Xu , Y. Xiong , M. H. Tang , B. W. Zeng , and Y. G. Xiao , “Bipolar and Unipolar Resistive Switching Modes in Pt/Zn_0.99_Zr_0.01_O/Pt Structure for Multi‐Bit Resistance Random Access Memory,” Applied Physics Letters 104 (2014): 183501.

[54] C. Y. Zhuge , J. D. Jiang , L. Chen , et al., “Subquantum Semimetal Bi and Oxygen Vacancy Filament Memristors for Neuromorphic Computing,” ACS Applied Materials & Interfaces 17 (2025): 34129–34138. 40457765 10.1021/acsami.5c02955

[55] L. Q. Zou , Z. R. Peng , H. J. Sun , et al., “Bio‐Realistic Synaptic‐Replicated “V” Type Oxygen Vacancy Memristor,” Advanced Functional Materials 35 (2025): 2416325.

[56] Y. Park , U. B. Han , M. K. Kim , and J. S. Lee , “Solution‐Processed Flexible Threshold Switch Devices,” Advanced Electronic Materials 4 (2018): 1700521.

[57] B. J. Dang , J. Sun , T. Zhang , et al., “Physically Transient True Random Number Generators Based on Paired Threshold Switches Enabling Monte Carlo Method Applications,” IEEE Electron Device Letters 40 (2019): 1096–1099.

[58] Y. R. Jeon , D. Akinwande , and C. Choi , “Volatile Threshold Switching and Synaptic Properties Controlled by Ag Diffusion Using Schottky Defects,” Nanoscale Horizons 9 (2024): 853–862. 38505960 10.1039/d3nh00571b

[59] I. Oh and J. J. Pak , “Coexistence of volatile and non‐Volatile Characteristics in SiO_2_/CoOx Memristor for in‐Materia Reservoir Computing,” Journal of Alloys and Compounds 1020 (2025): 179383.

[60] V. K. Sahu , A. K. Das , R. S. Ajimsha , and P. Misra , “Studies on Transient Characteristics of Unipolar Resistive Switching Processes in Tio_2_ Thin Film Grown By Atomic Layer Deposition,” Journal of Physics D: Applied Physics 51 (2018): 215101.

[61] R. Waser , R. Dittmann , G. Staikov , and K. Szot , “Redox‐Based Resistive Switching Memories—Nanoionic Mechanisms, Prospects, and Challenges,” Advanced Materials 21 (2009): 2632–2663. 36751064 10.1002/adma.200900375

[62] I. Valov , R. Waser , J. R. Jameson , and M. N. Kozicki , “Electrochemical Metallization Memories—Fundamentals, Applications, Prospects,” Nanotechnology 22 (2011): 254003. 21572191 10.1088/0957-4484/22/25/254003

[63] A. Chaves , J. G. Azadani , H. Alsalman , et al., “Bandgap Engineering of Two‐Dimensional Semiconductor Materials,” npj 2D Materials and Applications 4 (2020): 29.

[64] F. Zhang , H. R. Zhang , S. Krylyuk , et al., “Electric‐Field Induced Structural Transition In Vertical MoTe_2_ ^−^ and Mo_1–x_W_x_Te_2_‐Based Resistive Memories,” Nature Materials 18 (2019): 55–61. 30542093 10.1038/s41563-018-0234-y

[65] I. Valov , E. Linn , S. Tappertzhofen , et al., “Nanobatteries in Redox‐Based Resistive Switches Require Extension of Memristor Theory,” Nature Communications 4 (2013): 1771. 10.1038/ncomms2784 PMC3644102 23612312

[66] J. D. Lin , C. Han , F. Wang , et al., “Electron‐Doping‐Enhanced Trion Formation in Monolayer Molybdenum Disulfide Functionalized With Cesium Carbonate,” Acs Nano 8 (2014): 5323–5329. 24785254 10.1021/nn501580c

[67] C. Lee , H. Yan , L. E. Brus , T. F. Heinz , J. Hone , and S. Ryu , “Anomalous Lattice Vibrations of Single‐ and Few‐Layer MoS_2_ ,” Acs Nano 4 (2010): 2695–2700. 20392077 10.1021/nn1003937

[68] M. Rajapakse , B. Karki , U. O. Abu , et al., “Intercalation as a Versatile Tool for Fabrication, Property Tuning, and Phase Transitions in 2D Materials,” npj 2D Materials and Applications 5 (2021): 30.

