# Supplementary material for: Cation‐Driven Valence Change Mechanism in 2D AgCrS2 for Ultralow‐Power and Reliable Memristors
Source: Adv Sci (Weinh). 2026 Feb 4;13(18):e21409. doi: 10.1002/advs.202521409 (PMC13042405; doi:10.1002/advs.202521409)
Supplement: Supplementary file 1 — Supporting File 1: advs73952‐sup‐0001‐SuppMat.docx. [file ADVS-13-e21409-s002.docx]

Supporting Information

**Cation-Driven Valence Change Mechanism in Two-Dimensional AgCrS_2_ for Ultralow-Power and Reliable Memristors**

*Yueqi Su, Minghao Wang, Xiaolin Tai, Yuhua Liu, Yue Lin, Yuqiao Guo, Jing Peng^*^, Yi Xie, and Changzheng Wu^,*^*

**Experimental Section**

*Synthesis of AgCrS_2_ Nanosheets*: Single crystals of AgCrS_2_ were grown by chemical vapor transport (CVT) from the corresponding polycrystalline precursors using CrCl_3_ as the transport agent.^[1]^ AgCrS_2_ nanosheets were obtained from the exfoliation of bulk AgCrS_2_ crystals by utilizing a redox-controlled electrochemical exfoliation method.^[2]^ In details, a platelet of AgCrS_2_ was clamped between two titanium holders and connected to the cathode, while a platinum foil served as the anode. A 0.1 M solution of tetraoctylammonium in acetonitrile served as the electrolyte. The intercalation step was carried out at a constant bias of 5 V for 1 h controlled by a CHI760E system. The intercalated crystal was subsequently transferred into N,N-dimethylformamide (DMF) and delaminated into nanosheets by gentle hand shaking. The resulting dispersion was purified by centrifugation at 1,000 r.p.m. for 3 min and repeated washing/redispersion to remove large chuckles and residual electrolyte.

*Device Fabrication and Transport Measurements*: The dispersion of AgCrS_2_ nanosheets were drop-casted onto SiO_2_/Si substrates and promptly removed to leave isolated AgCrS_2_ nanosheets on the substrates. The nanosheets were then rinsed three times with isopropanol and baked in a vacuum oven at 80 °C for 1 h to remove residual solvent. Au (50 nm) and Ag (50 nm) contacts were sequentially patterned by electron-beam lithography (Raith Pioneer Two) followed by electron-beam evaporation (AdNaNotek, EBS-150). For faster throughput, AgCrS_2_ nanosheets can be also transferred onto pre-patterned Au electrodes and subsequently capped with Ag on the other side. Devices fabricated by either route displayed no obvious differences in the measured characteristics. All I–V characteristics and pulse measurements were performed using a Keithley 4200-SCS Parameter Analyzer and Keithley 2400 SMU under vacuum at room temperature. The automatic pulse measurement was achieved by ETMeS code. ^[3]^

*Material characterizations*: The optical images and videos were collected by Olympus BX51M. Raman spectra were acquired at room temperature using a LabRAM HR Evolution Raman System spectrometer with excitation light of 532 nm. Conductive atomic force microscopy (CAFM) analyses were performed using a Bruker Dimension Icon microscope in contact mode. Electron probe microanalyzer (EPMA) mappings and quantitative analyses were carried out using a Shimadzu EPMA8050G microanalyser. High-resolution transmission electron microscopy (HRTEM) were performed on the JEM 2100F. The high-angle annular dark-field scanning transmission electron microscopy (HAADF-STEM) images were obtained using JEOL JEM-ARM200F.

*Calculation method*: All the calculations were implemented with density functional theory (DFT) method, as performed by Quantum Espresso (QE) package^[4, 5]^ for DOS and NEB calculation and VASP for AIMD simulation. We used scalar-relativistic norm-conserving pseudopotentials^[6]^ with a plane-wave cutoff energy of 80 Ry. Grimme’s DFT-D3 scheme^[7]^ was utilized to include the correction of van der Waals interactions. The Brillouin zones of AgCrS_2_ bulk and monolayer are sampled by 16 × 16 × 16 and 6 × 6 × 1 mesh points in k-space based on Monkhorst-Pack scheme. The vacuum region was set to 15 Å in the perpendicular direction to the plane for the slab model of monolayer AgCrS_2_. The convergence criterion in self-consistent (SCF) is set to be 10^-8^ Ry, the force and stress convergence criterion for structure optimizations are set to be 10^-4^ Ry/Bohr and 0.1 kPa.


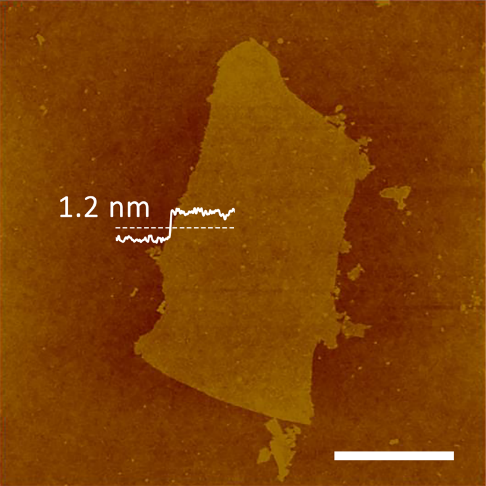


**Figure S1.** AFM image of a AgCrS_2_ monolayer sample. The inset shows the corresponding height profile along the dashed line. Scale bar, 2 μm.


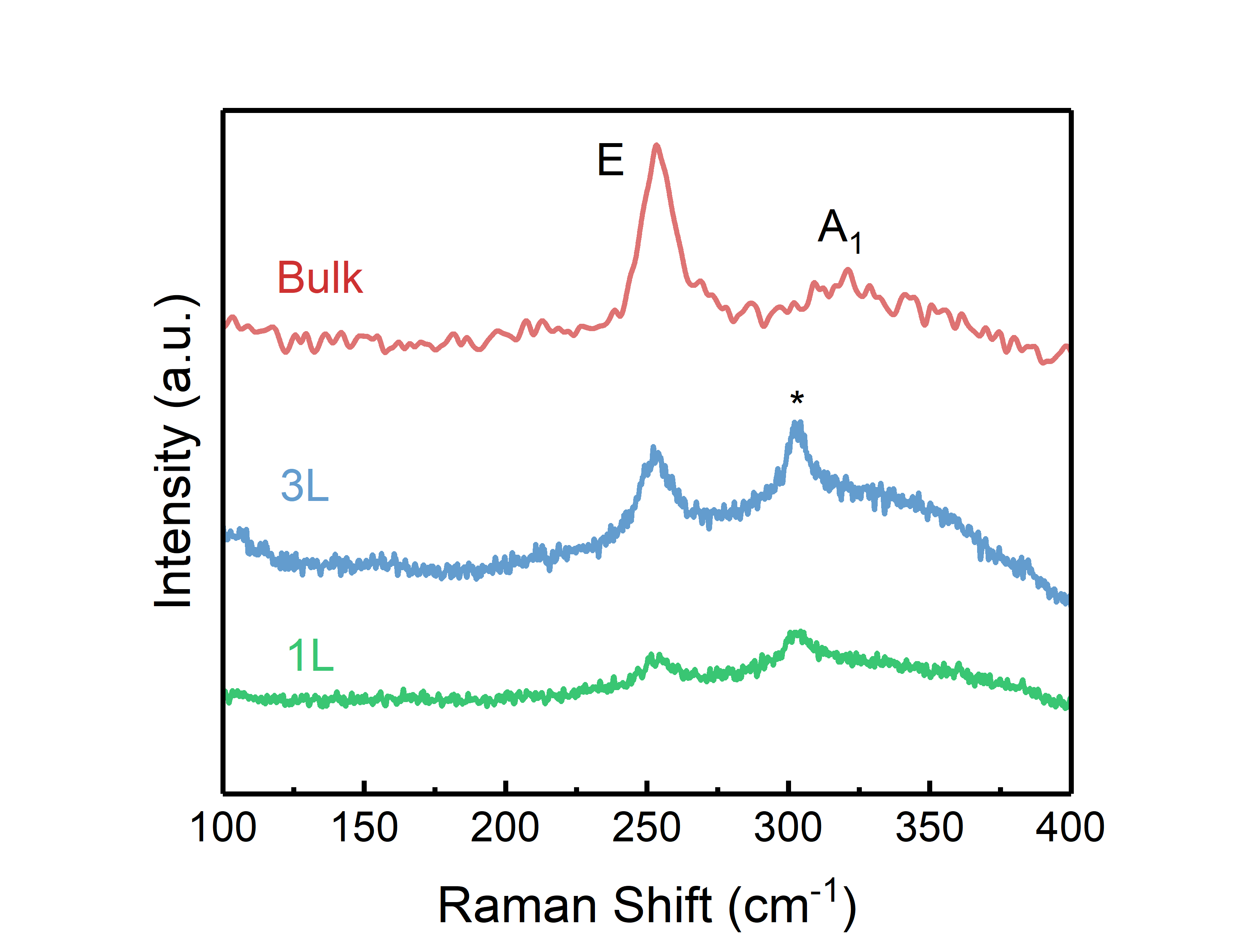


**Figure S2.** Raman spectra of AgCrS_2_ monolayer, trilayer and bulk material. The asterisk-marked peak is assigned to the silicon substrate.


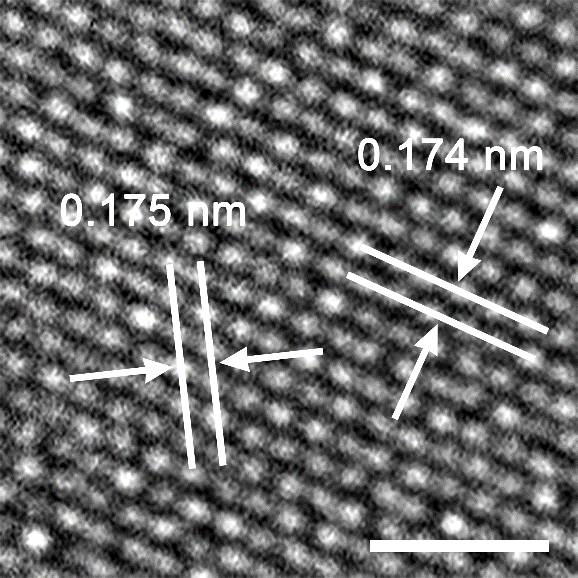


**Figure S3.** HRTEM image of AgCrS2 nanosheets. Scale bar, 1 nm.


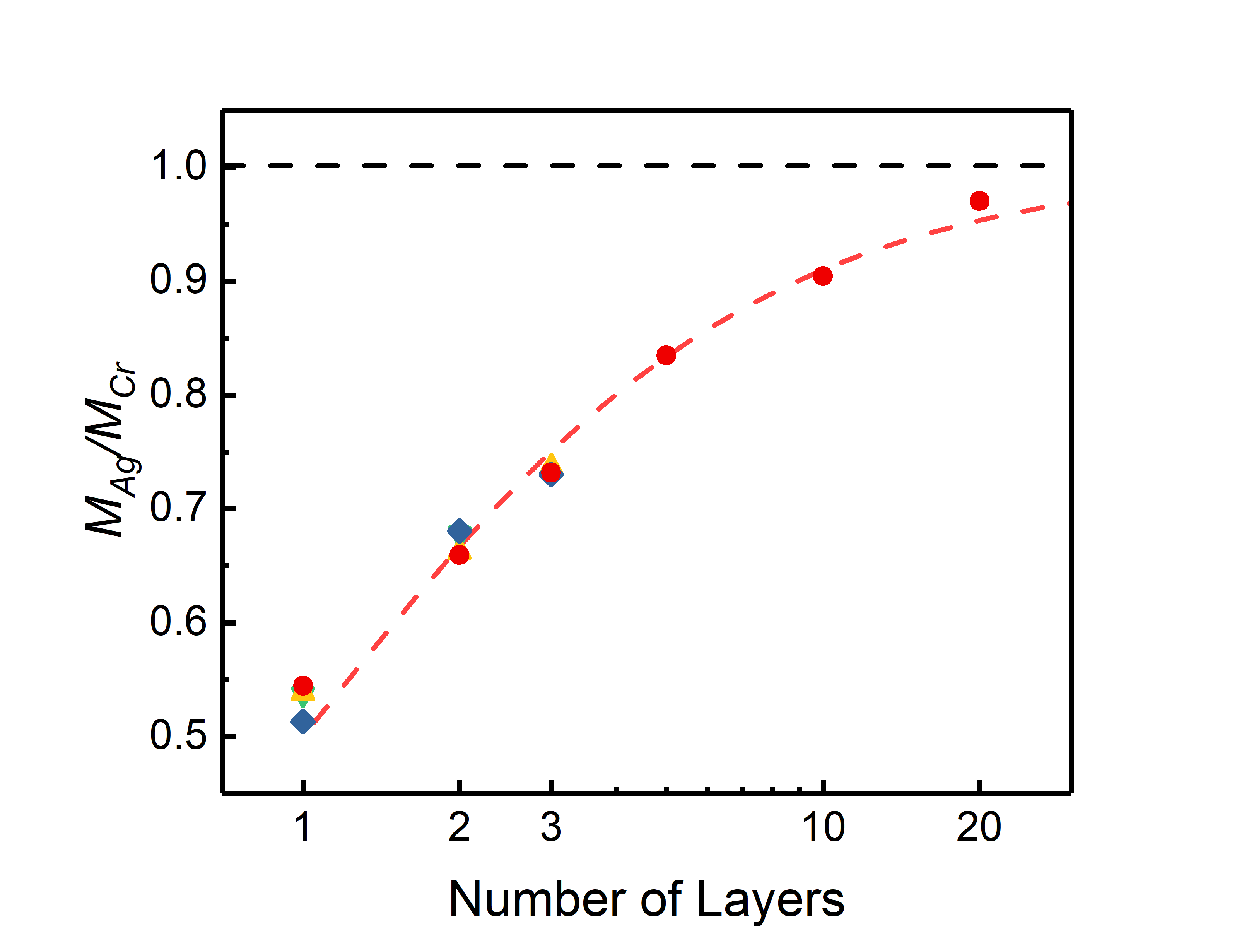


**Figure S4.** Molar ratio of Ag/Cr in AgCrS_2_ nanosheets with different numbers of layers. The red dashed line indicates the theoretical molar ratio of Ag/Cr for AgCrS_2_ nanosheets of different thicknesses.


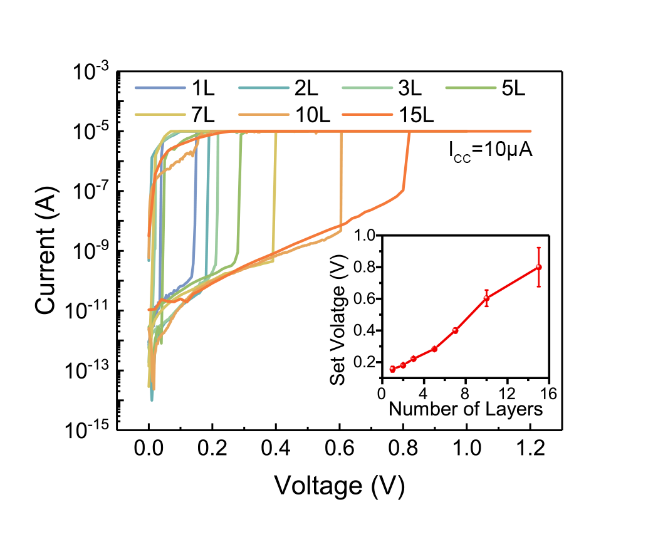


Figure S5. Switching behaviors of devices fabricated from AgCrS_2_ nanosheets with different thickness. The current compliance was set at 10 μA, The inset shows a thickness-dependent variation of the SET voltages.


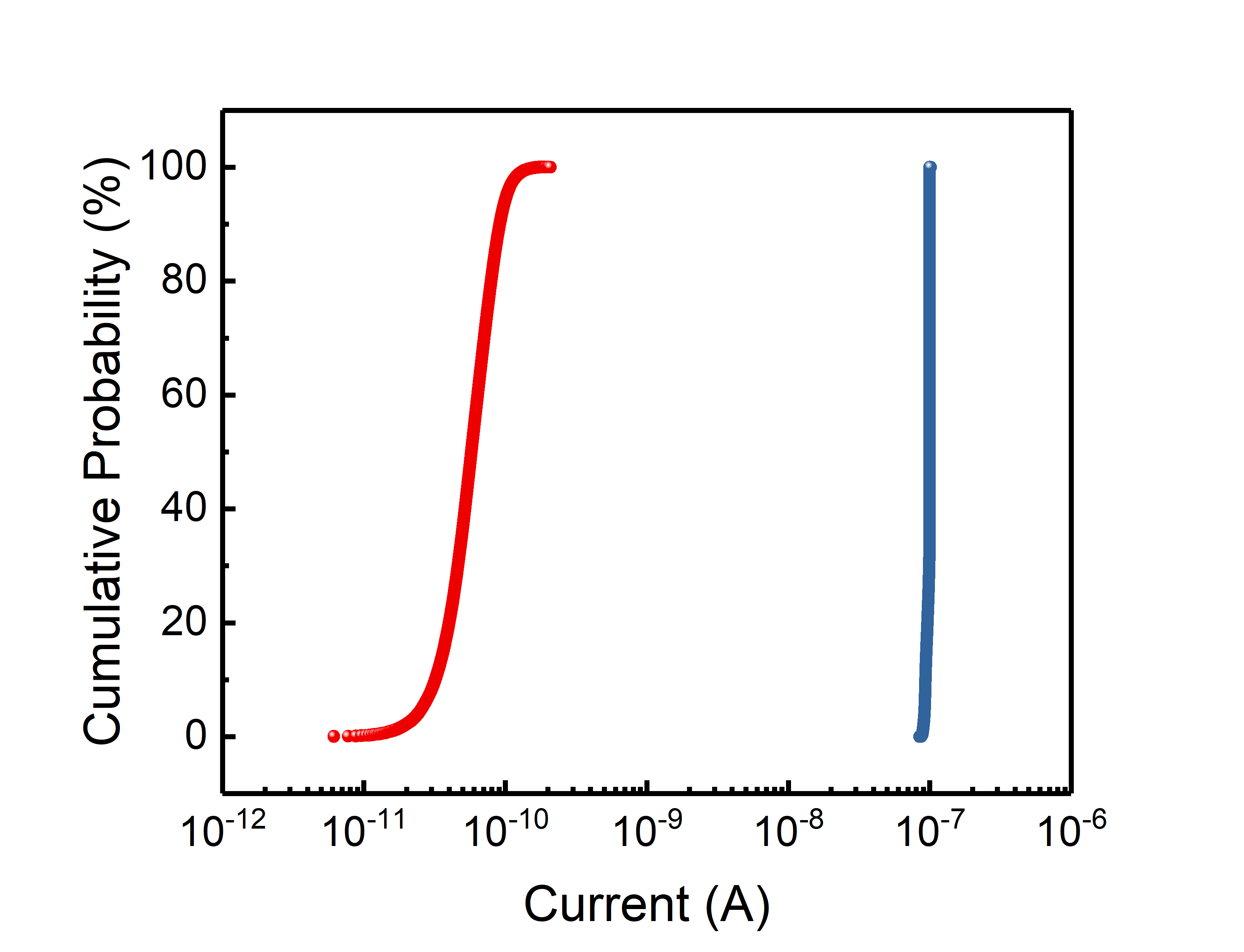


Figure S6. Cumulative probability of the LRS and HRS currents extracted from Figure 2f.


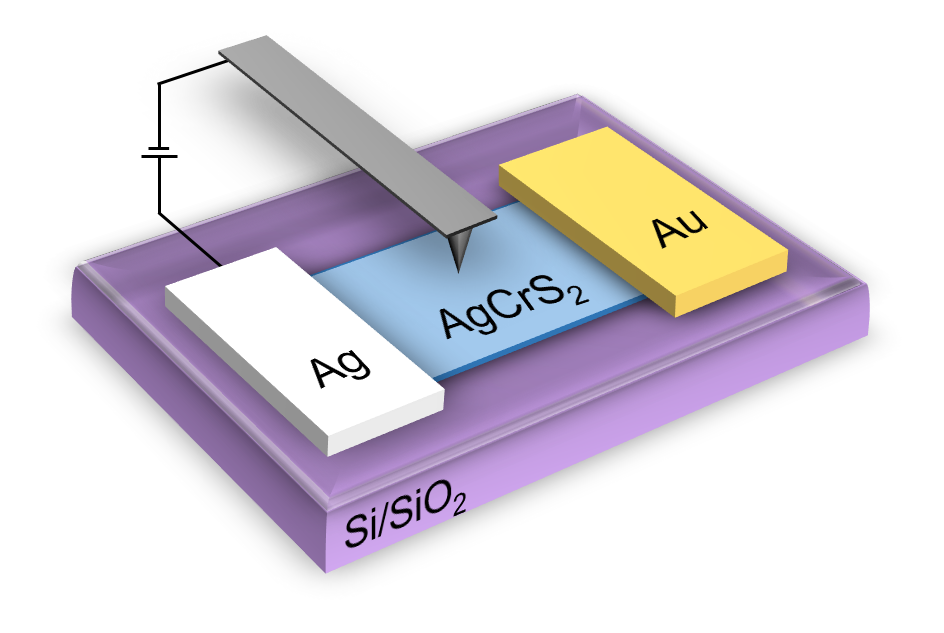


Figure S7. Schematic of the CAFM experimental setup.


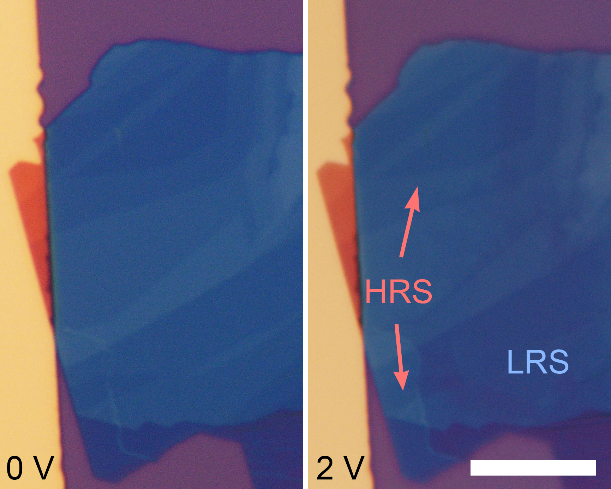


**Figure S8.** Optical images of the sample used for EPMA mapping. The colour switching indicates a transition from the HRS to the LRS in specific regions. Scale bar, 10 μm.


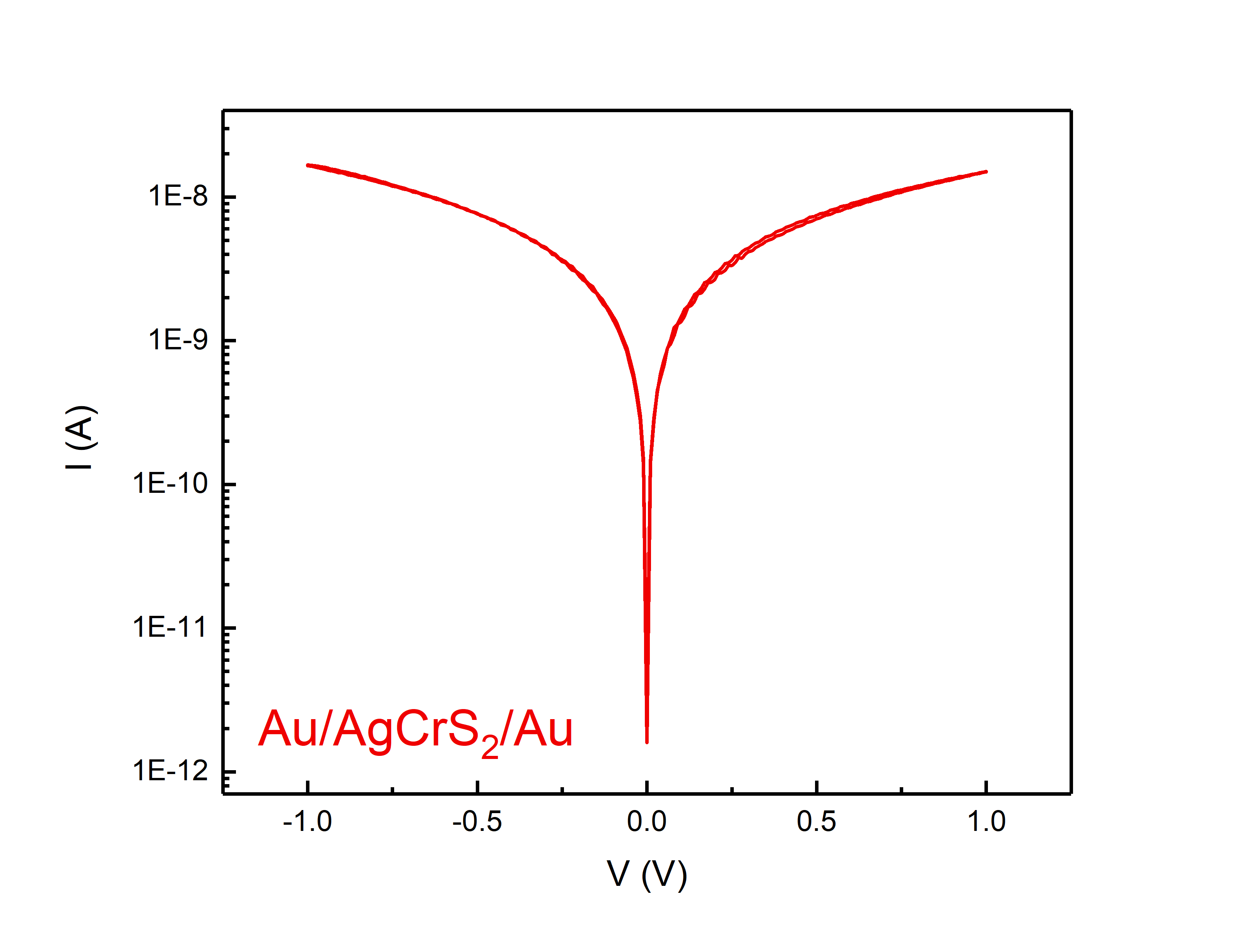


**Figure S9.** I-V curve of Au/AgCrS_2_/Au device. The curve shows no switching behavior, indicating that the resistive switching requires migration of external intercalated Ag^+^ cations instead of O^2-^ or S^2-^ anions.


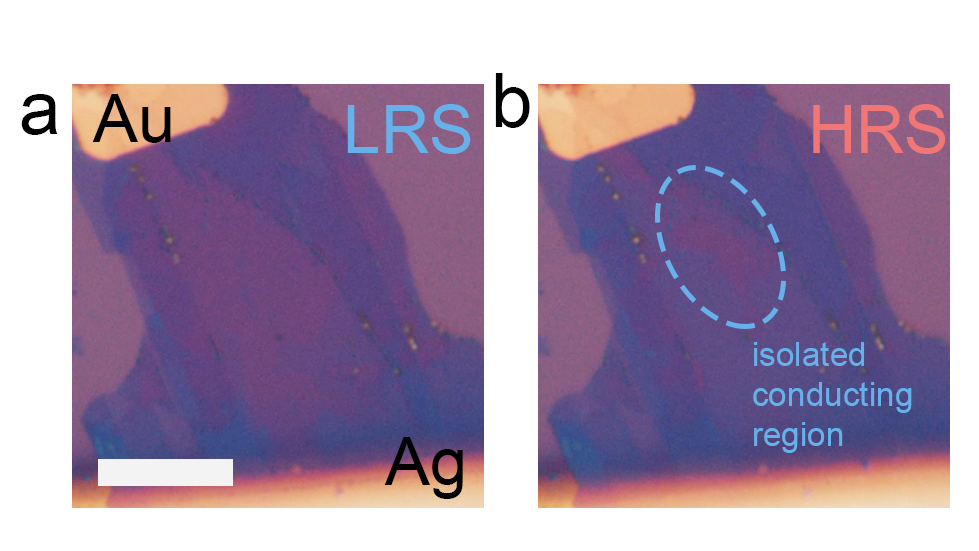


**Figure S10.** Optical images of the AgCrS_2_ device in the LRS (a) and 5 s after the voltage drops below V_HOLD_ (b). The continuous colorized pathway breaks into isolated colorized regions, indicating rupture of the conductive channel and relaxation of the device back to the macroscopic HRS.


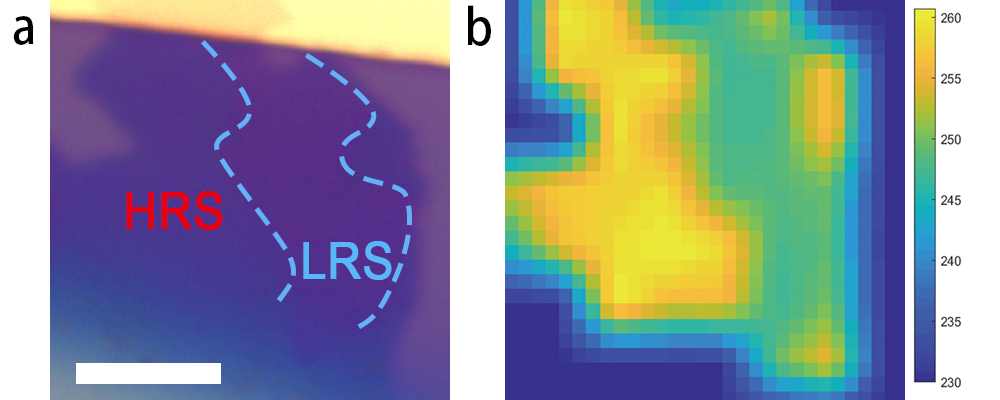


**Figure S11.** *In-situ* (**a**) optical image and (**b**) Raman intensity map of a sample in the LRS. The dashed outline in **a** indicates the LRS region, which corresponds to the area showing a redshift in the Raman E peak in **b**. Scale bar, 10 μm.


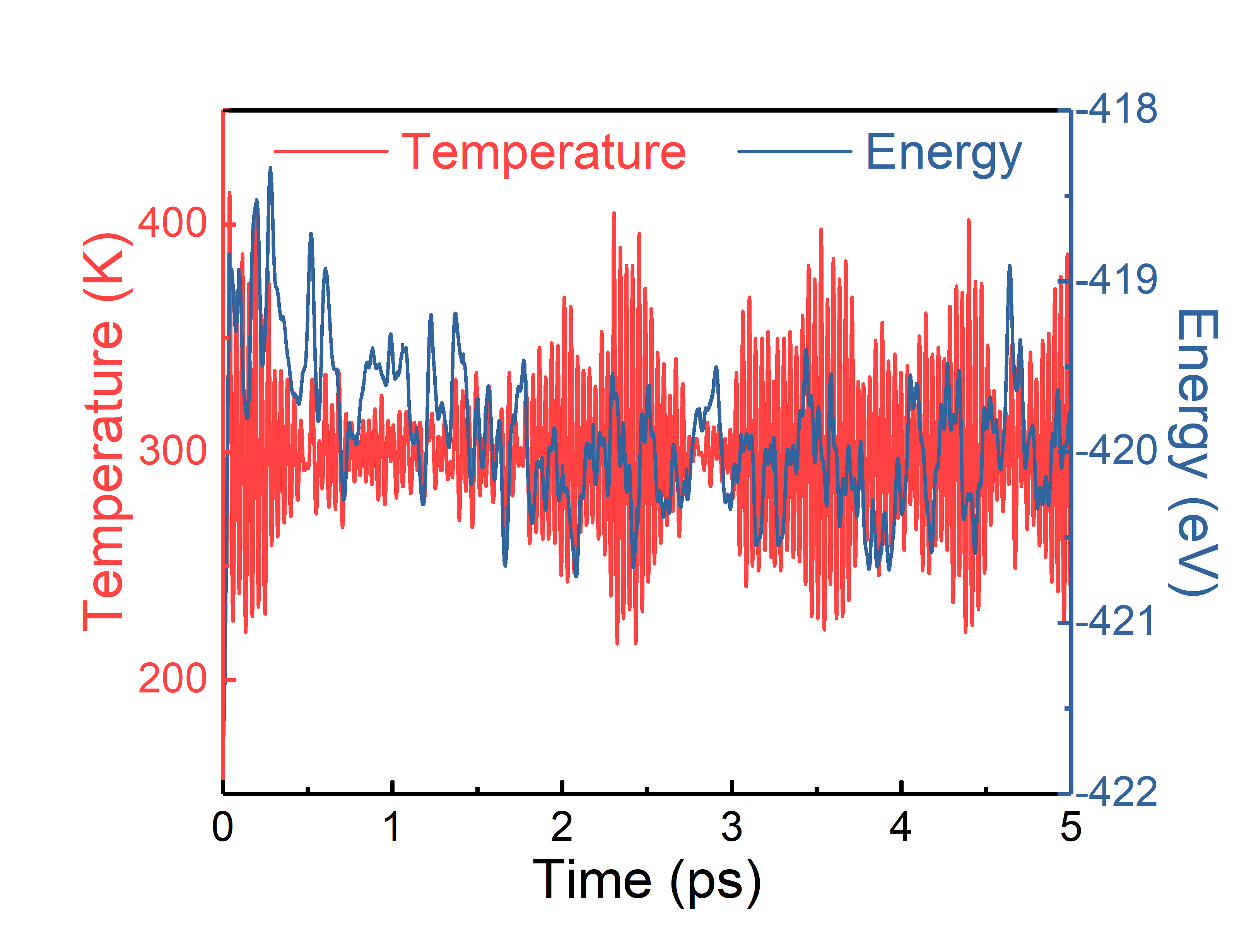


**Figure S12.** The fluctuation of temperature and total energy with time from the MD simulation of monolayer Ag_2_CrS_2_.

**References**

1. R. Yano, T. Sasagawa, *Cryst. Growth Des.* **2016**, 16, 5618.
2. J. Peng, Y. H. Liu, H. F. Lv, Y. X. Li, Y. Lin, Y. Q. Su, J. J. Wu, H. F. Liu, Y. Q. Guo, Z. W. Zhuo, X. J. Wu, C. Z. Wu, Y. Xie, *Nat. Chem.* **2021**, 13, 1235.
3. wmh137, *ETMeS*, 2025, https://github.com/wmh137/ETMeS.
4. P. Giannozzi, S. Baroni, N. Bonini, M. Calandra, R. Car, C. Cavazzoni, D. Ceresoli, G. L. Chiarotti, M. Cococcioni, I. Dabo, A. Dal Corso, S. de Gironcoli, S. Fabris, G. Fratesi, R. Gebauer, U. Gerstmann, C. Gougoussis, A. Kokalj, M. Lazzeri, L. Martin-Samos, N. Marzari, F. Mauri, R. Mazzarello, S. Paolini, A. Pasquarello, L. Paulatto, C. Sbraccia, S. Scandolo, G. Sclauzero, A. P. Seitsonen, A. Smogunov, P. Umari, R. M. Wentzcovitch, *J. Phys-condens. Mat.* **2009**, 21, 395502.
5. P. Giannozzi, O. Andreussi, T. Brumme, O. Bunau, M. B. Nardelli, M. Calandra, R. Car, C. Cavazzoni, D. Ceresoli, M. Cococcioni, N. Colonna, I. Carnimeo, A. Dal Corso, S. de Gironcoli, P. Delugas, R. A. DiStasio, A. Ferretti, A. Floris, G. Fratesi, G. Fugallo, R. Gebauer, U. Gerstmann, F. Giustino, T. Gorni, J. Jia, M. Kawamura, H. Y. Ko, A. Kokalj, E. Küçükbenli, M. Lazzeri, M. Marsili, N. Marzari, F. Mauri, N. L. Nguyen, H. V. Nguyen, A. Otero-de-la-Roza, L. Paulatto, S. Poncé, D. Rocca, R. Sabatini, B. Santra, M. Schlipf, A. P. Seitsonen, A. Smogunov, I. Timrov, T. Thonhauser, P. Umari, N. Vast, X. Wu, S. Baroni, *J. Phys-condens. Mat.* **2017**, 29, 465901.
6. D. R. Hamann, *Phys. Rev. B* **2013**, 88, 085117.
7. S. Grimme, J. Antony, S. Ehrlich, H. Krieg, *J. Chem. Phys.* **2010**, 132, Pmid 20423165.
